# Survey data on factors that drive farmers away from straw burning in the Mekong Delta, Vietnam

**DOI:** 10.1016/j.dib.2025.112318

**Published:** 2025-11-23

**Authors:** Ong Quoc Cuong, Matty Demont, Isabelita M. Pabuayon, Dinah Pura T. Depositario

**Affiliations:** a School of Economics, Can Tho University, Can Tho, Vietnam; b International Rice Research Institute (IRRI), Los Baños, Laguna, Philippines; c Department of Agricultural and Applied Economics, College of Economics and Management, University of the Philippines Los Baños, Los Baños, Laguna, Philippines; d Department of Agribusiness Management and Entrepreneurship, College of Economics and Management, University of the Philippines Los Baños, Los Baños, Laguna, Philippines

**Keywords:** Rice straw management, Straw burning, Removal, Incorporation, Environmental pollution, Mekong Delta, Vietnam

## Abstract

The dataset presents survey data on factors that drive farmers away from straw burning in the Mekong Delta, Vietnam. Data were collected in 2019 through face-to-face interviews with 543 farmers using a structured questionnaire in four provinces: Kien Giang, An Giang, Dong Thap, and Can Tho. The dataset includes variables related to socio-economic characteristics, production factors, and geographical location that influence farmers’ choices of rice straw management practices. Further analysis and interpretive insights are available in a related research article. These data are valuable for policymakers who can use these insights to design environmental policies that discourage rice straw burning, thereby reducing environmental pollution and mitigating greenhouse gas emissions. These data can also be used in broader studies that compare the factors influencing farmers’ choices of rice straw management in developing countries.

Specifications Table

**Table:** 

Subject	Agricultural Sciences
Specific subject area	Agricultural Economics, Sustainable Rice Straw Management
Type of data	CSV data file Raw, Analyzed
Data collection	Data collection was done through face-to-face interviews using a structured questionnaire in four provinces in the Mekong Delta. First, one district was selected in every province based on rice planted area and straw production. Next, two communes were selected in each district. Lastly, farmers were randomly selected in each commune. The research team developed a structured questionnaire to elicit information about rice straw management from farmers. Trained enumerators conducted the interviews, and field supervisors checked the completed questionnaires for completeness and consistency.
Data source location	City/Town/Region: Kien Giang (9.38°N**–**10.53°N Latitude and 103.50°E**–**105.53°E Longitude)**,** Dong Thap (10.12°N to 10.97°N Latitude and 105.20°E to 105.97°E Longitude), An Giang (10.17°N to 10.95°N Latitude and 104.77°E to 105.58°E Longitude), and Can Tho (9.92°N**–**10.33°N Latitude and 105.23°E**–**105.84°E Longitude) Country: Vietnam
Data accessibility	Repository name: Harvard Dataverse Survey data on factors that drive farmers away from straw burning in the Mekong Delta, Vietnam [1] Data identification number: 10.7910/DVN/MJK1L5 Direct URL to data: https://doi.org/10.7910/DVN/MJK1L5
Related research article	Cuong, O.Q., Demont, M., Pabuayon, I.M., Depositario, D.P.T. “What drives rice farmers away from straw burning? Evidence from the Mekong Delta, Vietnam”, Environmental Challenges (2025) 101,150. https://doi.org/10.1016/j.envc.2025.101150 [2]

## 1 Value of the Data

• The data enables the identification of factors that drive farmers away from straw burning.
• The data are useful for policymakers who can use these insights to design environmental policies that discourage rice straw burning, thereby reducing environmental pollution and mitigating greenhouse gas emissions.
• The data can be helpful to academicians and researchers interested in agricultural technology adoption and rice straw management.
• The data can be used as a baseline for assessing low-emission rice production programs and help evaluate the agricultural sector’s efforts to mitigate climate change at the national level.
• The data can be used for broader studies that compare the factors influencing farmers’ choices of rice straw management in developing countries.

## 2 Background

Burning of rice straw is a common practice in intensive rice cultivation systems in the Mekong Delta. The expansion of intensive rice cultivation in the Mekong Delta has the capacity to substantially augment the biomass of burned crop residues, thereby contributing significantly to GHG emissions [3]. To reduce air pollution from the burning of rice straw in Vietnam, alternative rice straw management practices, namely field incorporation of rice straw and partial removal, could be adopted.

This dataset presents a survey of rice farmers in the Mekong Delta, designed to capture the factors that drive them away from straw burning. Data were collected through face-to-face interviews using a structured questionnaire. The dataset contains information related to socio-economic characteristics, production factors, and geographical location of 543 farmers from four provinces in the Mekong Delta: Kien Giang, An Giang, Dong Thap, and Can Tho.

Policymakers can harness these insights to design and implement environmental policies that incentivize and scale up alternative rice straw management practices, thereby reducing pollution and mitigating greenhouse gas emissions. The data also serve as a valuable baseline for evaluation, as Vietnam advances its initiative to cultivate one million hectares of high-quality, low-emission rice, aligned with the country's green growth strategy in the Mekong Delta.

## 3 Data Description

The questionnaire and dataset are available as supplementary materials in the online version of this data article. The file “Data-Rice straw.csv” contains the data used in the analysis described in the article above. Table 1 summarizes the names of the variables as presented in the data set and their definitions.

**Table 1:** Definition of variables.

Variable Name	Definition	Answer/Coding
ID	Farmer identifier	1–543
Province	Province	1–Kien Giang; 2–Dong Thap; 3–Can Tho; 4–An Giang
RSMSA	Rice straw management in Summer-Autumn season	1–Burning of stubbles and/or loose straw; 2–Removal of loose straw and incorporation of stubbles; 3–Incorporation of loose straw and stubbles
RSMAW	Rice straw management in Autumn-Winter season	1–Burning of stubbles and/or loose straw; 2–Removal of loose straw and incorporation of stubbles; 3–Incorporation of loose straw and stubbles
RSMWS	Rice straw management in Winter-Spring season	1–Burning of stubbles and/or loose straw; 2–Removal of loose straw and incorporation of stubbles; 3–Incorporation of loose straw and stubbles
Age	Age of farmer	Years
Experience	Farming experience of farmer	Years
Education	Educational level of farmer	Years in school
Gender	Gender of farmer	1–Male; 0–Female
Area	Total area of farm land	Hectares
Income	Total annual gross household income	million VND
Organization	Membership in farmer organization	1–Member, 0–Otherwise
Training	Attendance in agricultural training	1–Attend, 0–Otherwise
Extension	Visit received from extension worker	1–Yes, 0–Otherwise
Credit	Access to credit from bank or relatives/friends	1–Yes, 0–Otherwise
Magazine	Reading agricultural magazines	1–Yes, 0–Otherwise
Contract	Having a production contract for paddy with a company	1–Yes, 0–Otherwise
Cultivation	Main rice cultivation practice	1–Modern, 0–Otherwise
KienGiang	Location of farm in Kien Giang province	1–Kien Giang, 0–Otherwise
DongThap	Location of farm in Dong Thap province	1–Dong Thap, 0–Otherwise
CanTho	Location of farm in Can Tho province	1–Can Tho, 0–Otherwise

Agricultural technology is a comprehensive concept that encompasses the development of agricultural inputs, farming practices, genetic materials, and equipment to enhance agricultural effectiveness [4]. Adoption is the incorporation of new technology into current practice, and is typically performed by a trial period and some degree of adaptation [5]. The factors that determine agricultural technology adoption are categorized as technological, economic, institutional, and household-specific factors [6]. Thus, the survey data contains the following: (i) rice straw management by season; (ii) socio-economic factors (age of farmer, farming experience of the farmer, educational level of the farmer, gender of the farmer, and total annual gross household income); (iii) production factors (total area of farm land, membership in farmer organization, attendance in agricultural training, contacting agricultural extension, access to credit, reading agricultural magazines, having a production contract for paddy with a company, and primary rice cultivation practice) and (iv) location (Kien Giang, Dong Thap, and Can Tho).

**Fig. 1:**
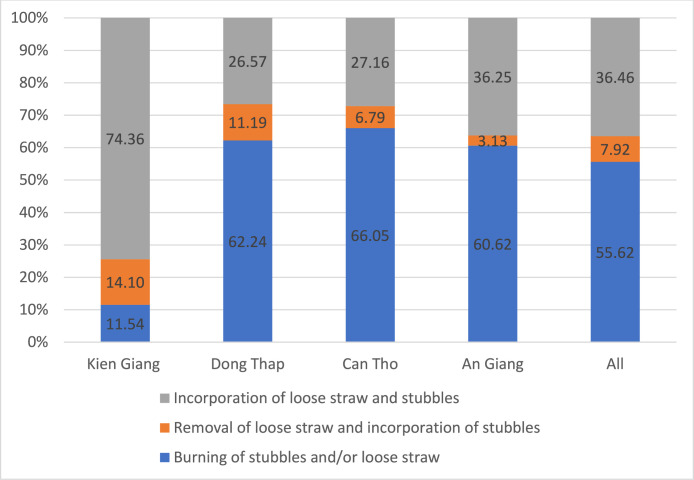
Rice straw management practices by province in the Summer-Autumn season.

**Fig. 2:**
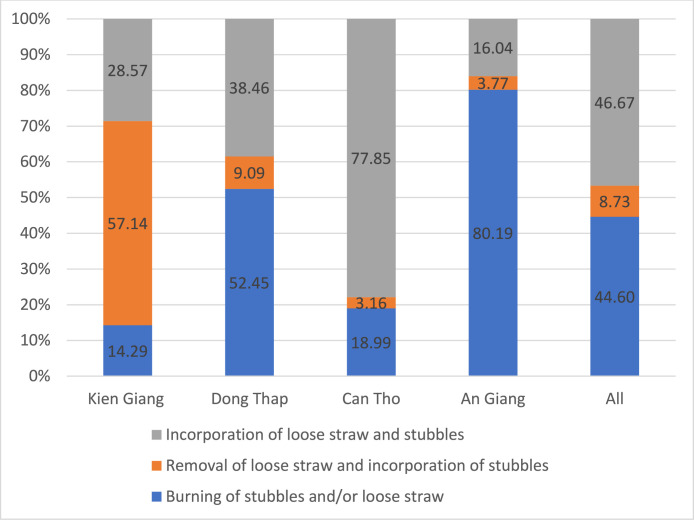
Rice straw management practices by province in the Autumn-Winter season.

**Fig. 3:**
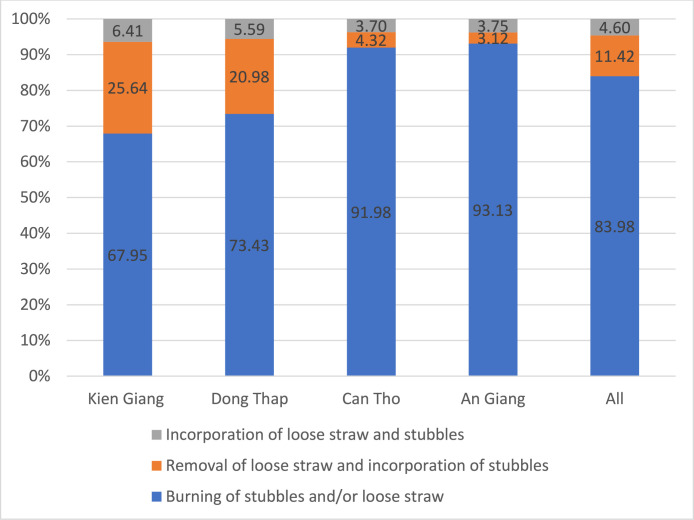
Rice straw management practices by province in the Winter-Spring season.

The available practices for rice straw management in the Mekong Delta are: (i) burning of stubbles and/or loose straw (Partial and Full burning), (ii) removal of loose straw and incorporation of stubbles (Partial removal), and (iii) incorporation of loose straw and stubbles (Full incorporation). The rice straw management practices by province are illustrated in Fig. 1, Fig. 2, Fig. 3.

## 4 Experimental Design, Materials and Methods

**Fig. 4:**
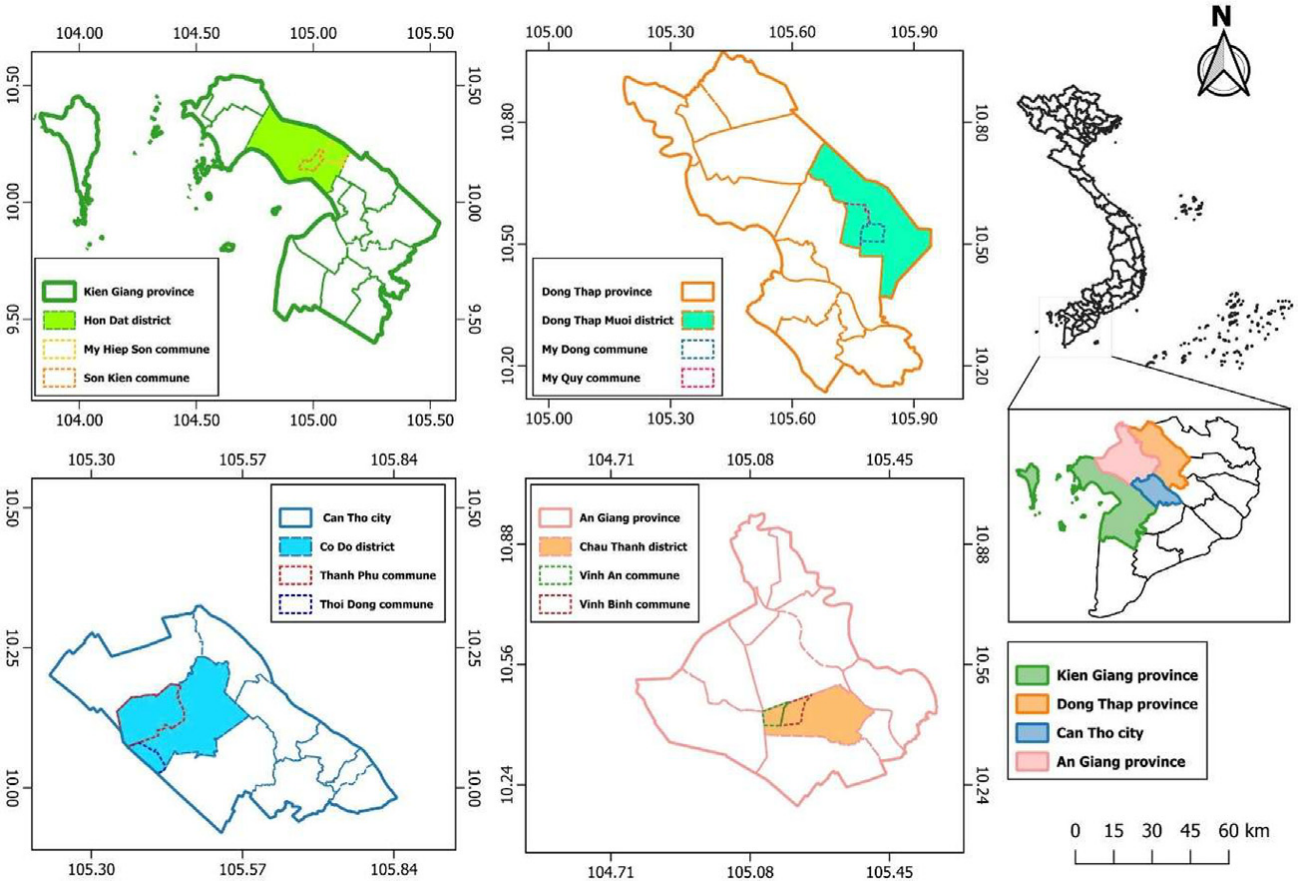
The geographical map of the study site.

The study area comprised four provinces in the Mekong Delta, Vietnam: Kien Giang, An Giang, Dong Thap provinces, and Can Tho city. Can Tho was considered to represent the economic center of the Mekong Delta. Three other provinces were purposively selected based on which had the highest production levels of rice straw. The geographical map of the study site is presented in Fig. 4.

Simple random sampling, the most basic method, provides every element of the sample frame with an equal chance of selection [7]. We used simple random sampling to select respondents from the selected provinces. The farming household was the study's unit of analysis. The first step was to select one district in each province, namely, the Hon Dat district in Kien Giang province, Dong Thap Muoi district in Dong Thap province, Co Do district in Can Tho city, and Chau Thanh district in An Giang province. Next, two communes were selected in each district, namely, Son Kien commune and My Hiep Son commune in Hon Dat district; My Dong commune and My Quy commune in Dong Thap Muoi district; Thoi Dong commune and Thanh Phu commune in Co Do district; and Vinh Binh commune and Vinh An commune in Chau Thanh district. Finally, we randomly selected households within each commune.

**Fig. 5:**
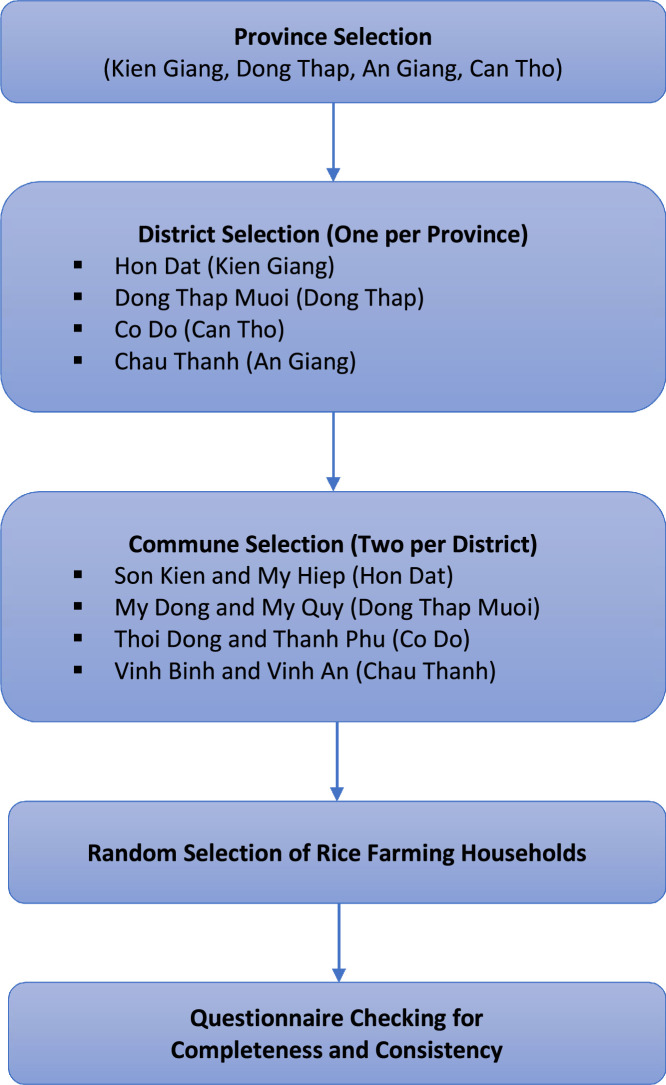
The flowchart of the sampling and data collection process.

The research team developed a structured questionnaire to elicit information about farmers’ rice straw management practices, as well as their socio-economic characteristics, production factors, and geographical location. The enumerators underwent one day of training on the structure of the questionnaire. The questionnaire was piloted in Hon Dat district, Kien Giang province. Whenever trained enumerators conducted the interviews, the field supervisors checked the completed questionnaires for completeness and consistency. The final sample consisted of 543 rice farmers across four provinces in the Mekong Delta: Kien Giang, An Giang, Dong Thap, and Can Tho. Fig. 5 illustrates the flowchart detailing the sampling and data collection process.

The structured questionnaire was comprised of a combination of dummy, categorical, and continuous variables aimed at capturing farmers’ socio-economic factors, production factors, and rice straw management practices. Dummy variables were coded using a 0–1 scheme, wherein 1 indicates the presence or participation (e.g., “membership in farmer organization”, “attendance of agricultural training”), while 0 denotes absence or non-participation. Additionally, dummy variables were constructed to represent farm location (e.g., Kien Giang, Dong Thap, and Can Tho) and the primary rice cultivation practice adopted by each farmer. This coding methodology enables consistent data processing and supports a range of statistical analyses, including descriptive statistics and hypothesis testing (i.e., *t*-tests and chi-square tests).

A multinomial logit model was used to determine the factors that influenced farmers’ rice straw management choices. STATA software (StataCorp LLC) was employed to estimate the model.

Future research could utilize this dataset to investigate farmers’ willingness to adopt alternative rice straw management practices under various policy scenarios, such as agricultural extension programs, monetary incentives, and carbon credits [8]. Additionally, it can serve as a baseline for assessing low-emission rice production programs and help evaluate the agricultural sector’s efforts to mitigate climate change at the national level.

## Limitations

This dataset presents some limitations. First, it covers only four provinces in the Mekong Delta—Kien Giang, Dong Thap, An Giang, and Can Tho—potentially restricting the applicability of the findings to other rice-cultivating areas in Vietnam. Second, the dataset primarily focuses on rice production and rice straw management, excluding data on other crops or livestock systems. Finally, the dataset captures socio-economic factors, production factors, and location, but does not include behavioral or psychological factors that may influence farmers’ decisions. Future surveys should incorporate these dimensions to provide a more thorough understanding of the factors influencing rice straw management practices.

## Ethics Statement

The survey was conducted in accordance with ethical standards and received approval from the International Rice Research Institute’s (IRRI) Institutional Research Ethics Committee (IREC no. 2019–0009-A-2015–117). Prior to each interview, the respondents were informed that: (i) the survey was for research purposes, (ii) their participation was voluntary, (iii) they were free to withdraw at any time, and (iv) all personal information would remain confidential. Informed consent was obtained from the respondents before proceeding with the interviews, and a copy of the consent form was provided to each respondent upon completion of the survey.

## CRediT Author statement

**Ong Quoc Cuong:** Conceptualization, Methodology, Formal analysis, Investigation, Data curation, Writing—Original draft, Writing—Review & Editing, Visualization. **Matty Demont:** Conceptualization, Methodology, Validation, Formal analysis, Investigation, Resources, Writing—Review & Editing, Visualization, Supervision, Project administration, Funding acquisition. **Isabelita M. Pabuayon:** Conceptualization, Methodology, Validation, Formal analysis, Writing—Review & Editing, Visualization, Project administration, Supervision. **Dinah Pura T. Depositario:** Conceptualization, Methodology, Writing—Review & Editing, Visualization.

## Data Availability

Harvard DataverseSurvey data on factors that drive farmers away from straw burning in the Mekong Delta, Vietnam (Original data).

## Appendix Supplementary materials

Image, application 1
