# Supplementary material for: Survey data on factors that drive farmers away from straw burning in the Mekong Delta, Vietnam
Source: Data Brief. 2025 Nov 23;64:112318. doi: 10.1016/j.dib.2025.112318 (PMC12881729; doi:10.1016/j.dib.2025.112318)
Supplement: Supplementary file 1 [file mmc1.docx]

**QUESTIONNAIRE**

**FACTORS THAT DRIVE FARMERS AWAY FROM STRAW BURNING**

**IN THE MEKONG DELTA, VIETNAM**

Respondent’s Name:

Address: Hamlet:

Commune: District:

Province:

Interview date:

Interview time:

1. Gender of farmer head: ❑ Male ❑ Female

2. Age of farmer head: years old

3. Educational level of farmer head: years in school

4. Farming experience of farmer head: years

5. Total area of farm land: hectares

6. Annual gross household income: 1,000 VND

7. Please confirm in which specific seasons below you cultivate rice:

a. Summer-Autumn 2018

b. Autumn-Winter 2018

c. Winter-Spring 2018-2019

8. What is your main rice cultivation practice? (Single answer)

a. Traditional rice cultivation

b. Three Reductions, Three Gains (3R3G)

c. One Must Do, Five Reductions (1M5R)

d. One Must Do, Six Reductions (1M6R)

e. Vietnamese Good Agricultural Practices (VietGaP)

f. Global Good Agricultural Practices (GlobalGAP)

g. Sustainable Rice Platform (SRP)

h. Others:

9. Do you have production contract for paddy with a company?

a. Yes ⇨ Q.10 b. No ⇨ Q.11

If yes, company name:

10. What is your kind of contract? (Single answer)

a. Marketing contract

b. Partial contract

c. Total contract

11. What is your main rice straw management practices in every cropping season? (Single answer)

| **Rice straw management practices** | **Summer**  **Autumn**  **2018** | **Autumn**  **Winter**  **2018** | **Winter**  **Spring**  **2018-2019** |
| --- | --- | --- | --- |
| a. Burning of loose straw and stubbles |  |  |  |
| b. Removal of loose straw and burning of stubbles |  |  |  |
| c. Removal of loose straw and incorporation of stubbles |  |  |  |
| d. Incorporation of loose straw and stubbles |  |  |  |

12. Are you membership in farmer organization?

a. Yes b. No

13. Do you attend in agricultural training?

a. Yes b. No

If yes, how many times do you attend in agricultural training? times/year

Please specify:

14. Whether or not an extension worker visited the farmer in the year? (contact with extension)

a. Yes b. No

15. Do you access credit from banks or your relatives/friends?

a. Yes b. No

If yes, when:

How much: 1,000 VND

16. Do you read agricultural magazines?

a. Yes b. No
